# Brachial artery aneurysm after hemodialysis fistula ligation: Case reports and review of literature

**DOI:** 10.1016/j.ijscr.2024.109306

**Published:** 2024-01-26

**Authors:** M.A. La Marca, E. Dinoto, E. Rodriquenz, F. Pecoraro, D. Turchino, D. Mirabella

**Affiliations:** a Vascular Surgery Unit - AOUP Policlinico ‘P. Giaccone’, Palermo, Italy; b Department of Surgical, Oncological and Oral Sciences – University of Palermo, Italy; c Department of Public Health, Vascular Surgery Unit, University Federico II of Naples, Italy

**Keywords:** Brachial artery aneurysm, Complication fistula, Renal transplantation, Dialysis, Surgical bypass

## Abstract

**Introduction:**

Brachial artery aneurysm (BAA) following long-standing arteriovenous fistula (AVF) ligation after renal transplantation is odd.

**Case presentation:**

Two cases of brachial artery aneurysm treated with bypass (a saphenous vein graft and a PTFE graft). In the first patient no complications were recorded whereas an infection was diagnosed after 6 months from the procedure in the second treatment.

**Clinical discussion:**

Multiple factors activated by stress on the vessel wall followed by fistula ligation are the cause of vascular remodeling of the three layers making up the wall with possible evolution in aneurysmatic lesions. In literature the gold standard for this lesion is the surgical approach, only one endovascular procedure is reported. The traditional surgical approach uses the autologous vein or prosthetic PTFE grafts.

**Conclusion:**

Brachial artery aneurysm is a complication that affects patients undergoing renal transplantation who have already undergone AVF ligation. In our experience autologous vein graft represented the best solution.

## Highlights

• Brachial Artery Aneurysm is a rare complication diagnosed often in renal transplant patients after closure of fistula.
• The main factor responsible is a stress on the vessel wall with endothelial injuries.
• The gold standard treatment is a surgical approach, only one endovascular procedure is reported in literature.
• The autologous vein graft represented the best solution.

## 1 Introduction

BAA is defined as a dilatation of the artery having a diameter greater than 10 mm or an increase of diameter greater than 50 % of the longitudinal axis. BAA is a rare complication (incidence of 4.5 %) diagnosed often in renal transplant patients, treated with closure of the hemodialysis' fistula with an incidence of 4.5 % [1]. Possible complications are peripheral embolization, thrombosis of the sac, rupture and hand ischemia [[2], [3], [4], [5], [6]]. The normal diameter of the brachial artery in women is 3.5–4.3 mm, while in men 4.1–4.8 mm [2]. True aneurysms occur in 0.17 % of cases, but usually are secondary to injury, infection and congenital defects. Data from the literature show that aneurysm after fistula ligation is a rare event with a median time from fistula ligation to aneurysm development of 120 months [5]. Here, we report our experience with two cases of a huge brachial artery aneurysm after fistula ligation with review of the literature.

This work has been written in accordance with the SCARE criteria [7].

## 2 Case 1

**Fig. 1:**
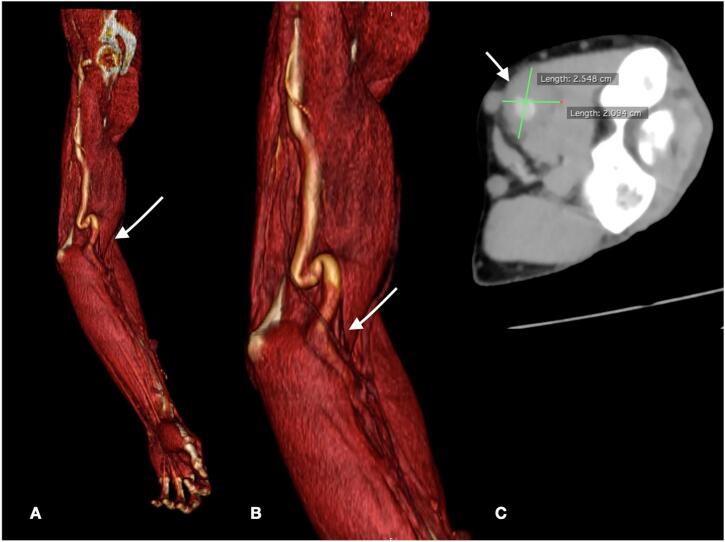
CT Angiography 3-dimensional volume rendering showing left arm (A) with tortuosity and extension of brachial aneurysm (B). CT Angiography with maximum diameter of aneurysm.

**Fig. 2:**
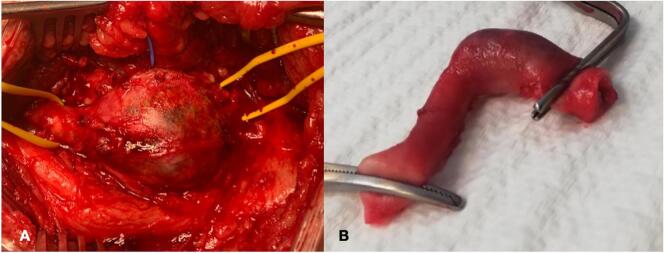
Intraoperative brachial aneurysm before (A) and after aneurysmectomy (B).

**Fig. 3:**
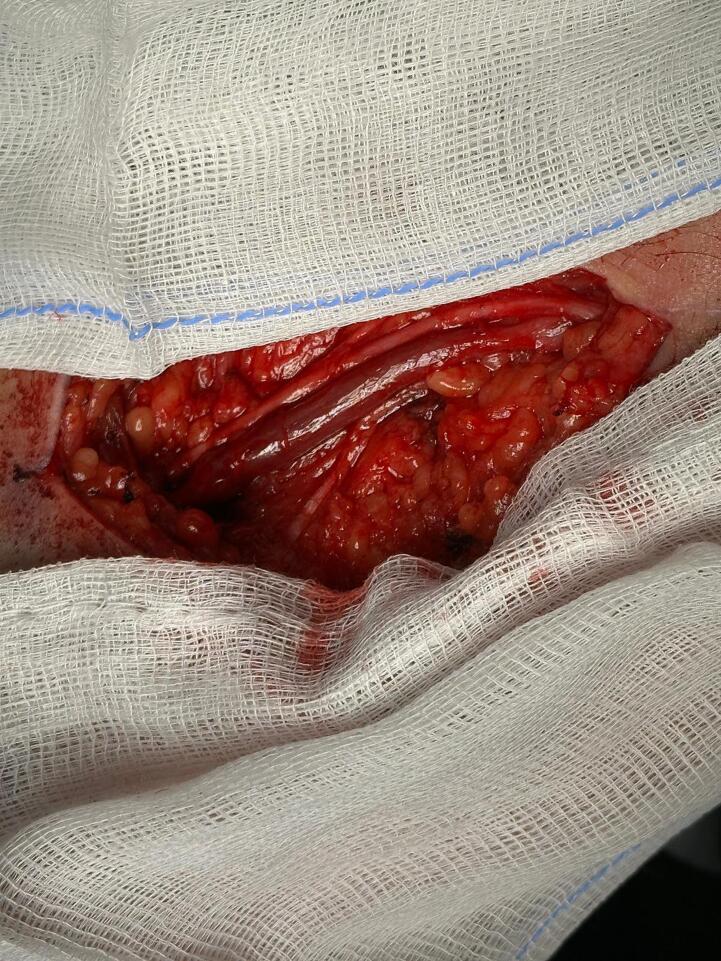
Intraoperative image of Bypass in vein after aneurysmectomy.

A 67-year-old man, referred to our hospital for a pulsating mass on the left forearm present from several months with increasing paresthesias and pain. CT angiography showed an aneurysmal dilatation of the brachial artery (25.5 mm × 19 mm) (Fig. 1). During the admission physical examination performance a medial pulsatile mass was shown on the ante cubital face of the left forearm. Axillary and brachial pulses were noticable to palpation, no radial flow was present due to thrombotic occlusion. Medical history reported terminal uremia, hypertension, previous gallbladder stones, cervical and lumbosacral spondylarthrosis. No-smoking patient, underwent left radio-cephalic AVF for chronic renal failure (CRF) due to IgA-deposited glomerulopathy, cadaver kidney transplantation in 2017 and subsequent fistula deafferentation. The patient's medication history includes immunosuppressive therapy with mycophenolic acid 360 mg and Tacrolimus, antiplatelet, antihypertensive and hypouricemic therapy. Despite the presence of CRF (creatinine 1.77 mg/dl preoperatively, GFR 38.9 ml/min), the patient did not required hemodialytic therapy. With the patient under supraclavicular block nerve anesthesia, a longitudinal incision was made in the bicipital groove as a way to expose the brachial artery until its terminal tract (Fig. 2). The aneurysm was isolated and sectioned at the ends in order to perform the aneurysmectomy. A brachial-brachial bypass using interposed reverse right saphenous was done (Fig. 3). Endovascular treatment was excluded due to thrombosis of the radial artery and the presence of neurological symptoms by compression on the median nerve. After treatment, no pain, edema or other significant symptoms was noticed and post-surgical therapy consisted of oral antibiotic (amoxicillin + clavulanic acid and LMWH). No complications were noticed during hospitalization and the patient was discharged on the 6th post-operative day. Follow-up lasted 9 months and was conducted with US scan which showed the long-term patency of the bypass.

## 3 Case 2

**Fig. 4:**
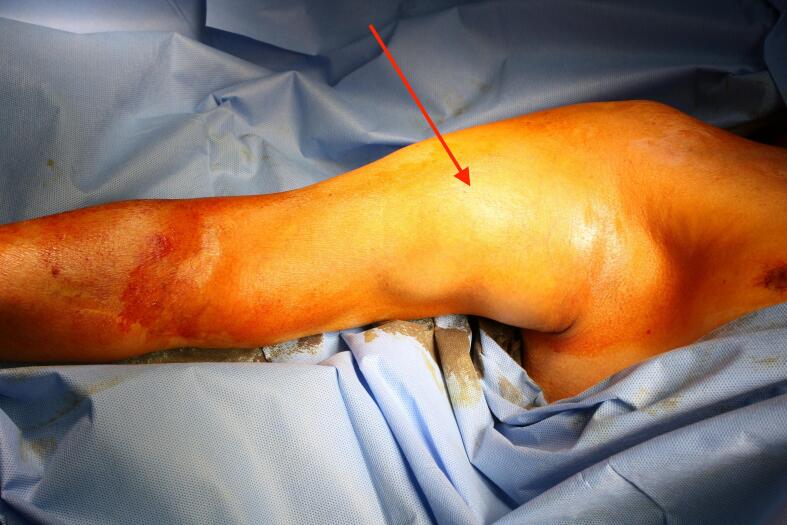
Proximal brachial aneurysm before surgical procedure.

**Fig. 5:**
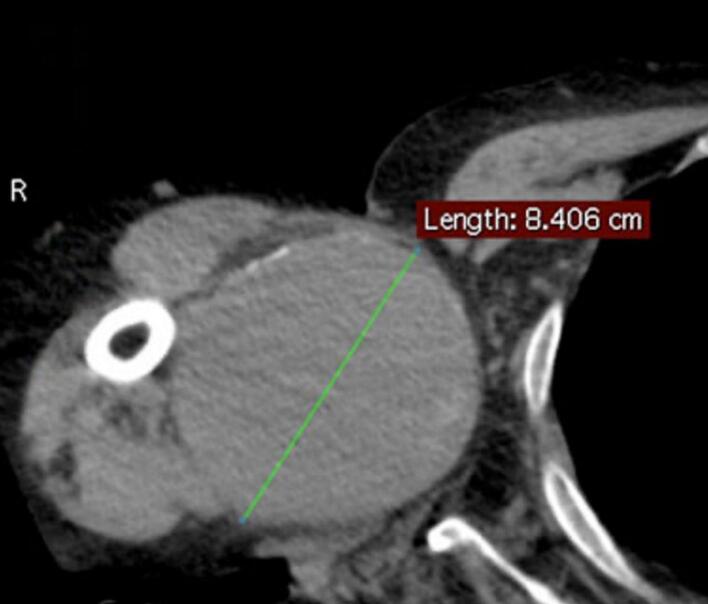
Basal CT scan showing huge brachial artery aneurysm with maximum diameter of 8.4 cm.

**Fig. 6:**
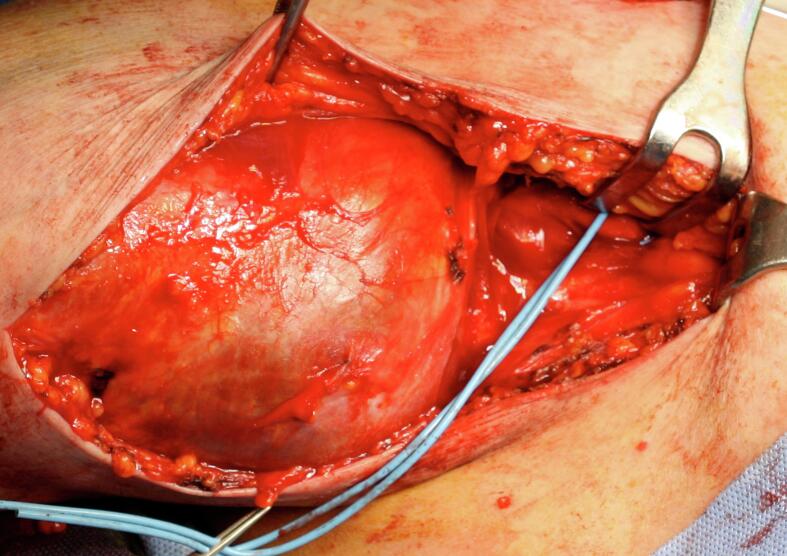
Intraoperative brachial aneurysm.

**Fig. 7:**
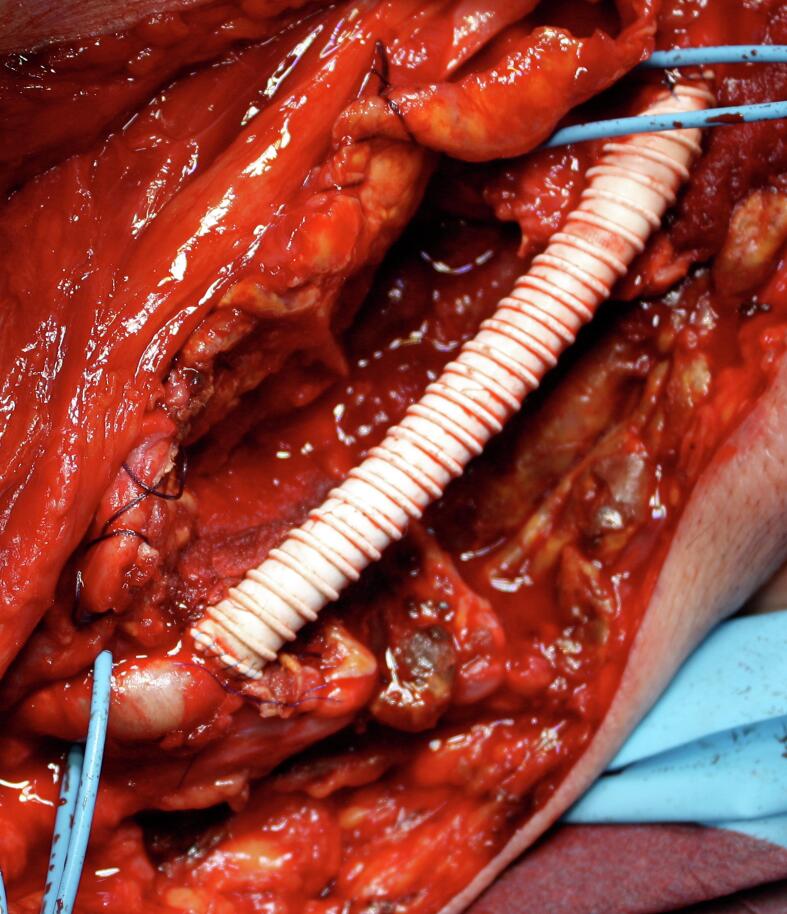
Intraoperative image of Bypass in PTFE after aneurysmectomy.

**Fig. 8:**
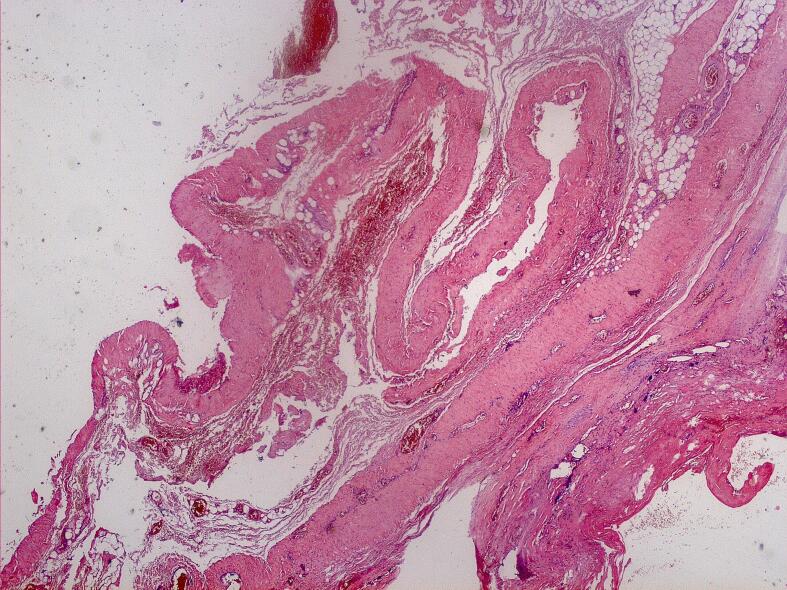
Histopathological features of the resected aneurysm. Hema- toxylin and eosin (H&E) stain at 925 magnification showing true aneurysm without infection/cells associated with an acute inflammatory process.

A 64-year-old man came to our attention for a progressive increase in the volume of right arm associated with pain and paresthesia in ipsilateral hand during the last month. On clinical examination, a large pulsatile mass was observed in a medial position towards the bicipital groove (Fig. 4). A duplex scan revealed an aneurysm approximately 8 cm in diameter. The patient had a history of renal failure due to IgA nephropathy and, as a result, he started hemodialysis therapy via a Cimino–Brescia AVF when he was 30 years old. The AVF was ligated after a successful cadaveric renal transplant which he underwent 13 years later. His past medical history included type 2 diabetes, hypertension, chronic obstructive pulmonary disease, cirrhosis due to chronic hepatitis C, and deforming arthrosis of the left hip treated with a hip replacement when he was 54 years old. Ever since he underwent renal transplantation, the patient had been on steroids and immunosuppressive drugs to prevent biocompatibility problems. Because of a history of allergy to contrast agent that had caused a severe respiratory crisis, the patient underwent a computed tomography (CT) scan without contrast. The images confirmed the presence of a BAA measuring 8.4 × 14 cm (Fig. 5). Endovascular treatment was not indicated because of the patient's allergy and the large size of the aneurysm, which caused symptoms of nerve compression. The aneurysm was therefore treated surgically. With the patient under general anesthesia, a longitudinal incision was made in the bicipital groove to expose the brachial artery (Fig. 6). The aneurysm was isolated and an incision was made in the aneurysm wall. A large concentric thrombus was removed. Since an autologous vein was not available as prosthetic material, a reinforced polytetrafluoroethylene (PTFE) prosthesis was used to re-establish blood flow in the arm (Fig. 7). A duplex scan performed in the postoperative period showed that the prosthetic graft was patent and that there was no peri-anastomotic stenosis. Histological examination using hematoxylin and eosin stain of the aneurysm wall showed that it consisted of intimal and medial degeneration with fibrous and inflammatory chronic tissue without signs of infection (Fig. 8). No complications were observed on clinical examination and duplex scanning performed 6 months after surgery but after 8 months an infection of prosthesis was diagnosed with wound dehiscence and graft explant in absence of ischemic symptoms for possible compensation coming from brachial profunda artery. The lesion was treated by closure for secondary intention.

## 4 Clinical discussion

The first case of brachial artery aneurysm was described by Hunter in 1757 and today 49 cases are reported in the literature [8]. Several factors are responsible of occurrence of brachial artery aneurysm after AVF ligation. According to Eugster et al. the main factor responsible is a stress on the vessel wall because of an increased blood flow, which causes the release of endothelial factors, such as nitric oxide, and an increase in vessel wall resistance resulting from fistula ligation [9]. Texeira et al. identify the etiology of lesion in an up-regulation of vasodilatory factors, degradation of elastic fibers and deposition on the vessel wall of calcium and phosphate secondary to dialysis, causing an activation of a pro-inflammatory process, in addition to immunosuppression and long-term corticosteroid therapy [10]. Carrol et al. describe other factors that can cause an inflammation of the vessel wall, such as hemodialysis, diabetes, dyslipidemia, hypertension, infection, and repeated trauma [11]. The use of long-term immunosuppressive therapy is another determinant of brachial artery aneurysm because it results in vascular remodeling of the three layers of the vessel wall due to the establishment of a cytokine-mediated inflammatory process [12]. Diagnostics uses different techniques such as duplex ultrasonography, computed tomography, magnetic resonance imaging, and digital subtraction angiography [[13], [14], [15], [16]]. Regarding AVF ligation after transplantation, some authors advise against it if the fistula is functioning and should be reserved for hand ischemia cases, risk of pseudo-aneurysm rupture, severe venous hypertension or significant high-output heart failure [17,18]. In literature non significative data withstand the role of fistula's position in BAA etiopathogenesis although most of the cases were related to AVF with cephalic vein (Table 1).

**Table 1:** Closed AVFs in patients with BAA reported in the literature.

Type of AVF	N. patient
Brachio-cephalic	21
Radio-cephalic	20
Undefined	6
Brachio-basilic	1
Brachio/radio-basilic	1

The gold standard for this lesion is a surgical approach with vein apposition when is possible thanks to the highest patency rate demonstrated in literature [[19], [20], [21]]. Only one case is reported with success as endovascular procedure where Maynar R. et al. treated a long BAA through the placement of several endoprosthesis in order to avoid a surgical approach because the patient was deemed “a poor candidate for surgical intervention due to the length of the aneurysm” [22].

The traditional surgical approach makes use of autologous veins and prosthetic PTFE grafts [[2], [3], [4]]. About a review of the literature, of the 49 reported cases of brachial artery aneurysm secondary arteriovenous fistula ligation, saphenous vein inverted was used in 21 patients (42.8 %), PTFE prostheses in 9 patients (18.4 %), cephalic vein or basilic vein inverted in 3 patients (6.2 %), other surgeries in 16 patients (32.6 %) (Table 2). In our experience the bypass in PTFE was complicated by an infection probably due to the lack of tissues necessary to protect a graft positioned less deeply than the skin, but in absence of adequate vein grafts for replacement, PTFE is a valid option [[23], [24], [25], [26]].

**Table 2:** Cases of BAA reported in the literature.

References	Age/sex	Clinical presentation	Graft	Aneurisma size (cm)	N.
Soares et al. [1]	66/M	Pain and swelling	VGS inverted	4.4	1
Dinoto et al. [2]	64/M	Pulsing swelling with discomfort and arm pain	PTFE protesis	8.4	1
Anastasiadou C et al. [13]	49/M	Pain and swelling	Cephalic vein	3.7	1
Barac et al. [27]	50.5/M	Pain and swelling	Varius (VGS inv, dacron graft)	6.5	2
Satoshi T et al. [28]	60/M	Pain and swelling	end-to-end anastomosis	3.5	1
Ferrara et al. [29]	61/M	Pain	Basilic Vein	N/a	1
Nguyen et al. [30]	51/M	Brachial and axillary pulse with a palpable supraclavicular thrill in the absence	VGS inverted	N/a	1
Battaglia et al. [31]	N/a	Pain	PTFE protesis	5	1
Murphy et al. [32]	61/M	Swelling in arm with some discomfort with rapid increase of swelling and pain over the 2 weeks preceding arrival at the unit	Cephalic vein	5	1
Chemla et al. [33]	N/a	Pain	VGS inverted	N/a	5
Basile et al. [34]	55/M	Development of a pulsatile swelling above the elbow	VGS inverted	2.4	1
Marzelle et al. [35]	38.1/7 M-3F	Pain and swelling	Varius (VGS ins 2, Basilic Vein 1, PTFE 3, Allograft 1, Dacron 1)	5.6	8
Garza et al. [36]	N/a	Pain and swelling	VGS inverted	N/a	1
Bahia et al. [37]	62.5/M	Pain with ischemia	VGS inverted	N/a	2
De Santis et al. [38]	47/M	Pain	Plastic of arterial wall	N/a	1
Khalid et al. [39]	N/a	Pain and swelling	VGS inverted	N/a	3
Gardiner et al. [40]	51.2 12 M-1F	Pain and swelling	Varius Vein conduit	N/a	13
Fernandez et al. [41]	49.5/M	Pain and swelling	VGS inverted	4.5	2
Correia et al. [42]	47/M	Asymptomatic	PTFE protesis	4.5	1
Rodrigues et al. [43]	54/F	Pain (rupture)	PTFE protesis	4.1	1
Lee et al. [44]	51/M	Pain and swelling	VGS inverted	3	1
Frandri J et al. [45]	26	Pain and swelling	Arterial trasposizione	N/a	5

N/a Not available.

## 5 Conclusions

Brachial artery aneurysm is a complication linked to patients undergoing renal transplantation who already sustained an AVF ligation. Several factors may predispose patients to the development of this complication, such as immunosuppressive therapy, diabetes, high blood pressure, and long-term hemodialytic replacement therapy. The choice of treatment in these patients is still traditional surgical approach, there is only one case available in literature about a patient treated with endovascular technique and even if it was reported as a complete success we have no evidence about its efficacy in the medium and long term on a significant number of patients. In our experience autologous vein graft represented the best solution.

## Institutional review board statement

All procedures followed were in accordance with the ethical standards of the Institutional Committee on Human Experimentation and with the Helsinki Declaration. According to the internal review board, the retrospective and anonymized nature of the study did not require medical ethical committee approval.

## Informed consent statement

Written informed consent was obtained from the patient for publication of this case report and accompanying images. A copy of the written consent is available for review by the Editor-in-Chief of this journal on request.

## Ethical approval

All procedures followed were in accordance with the ethical standards of the Institutional Committee on Human Experimentation and with the Helsinki Declaration. According to the internal review board, the retrospective and anonymized nature of the study did not require medical ethical committee approval.

This article does not require ethics committee approval due to the retrospective nature of the paper and the type of technique reported.

## Funding

This research received no external funding.

## Author contributions

Conceptualization, MA.L., E.D. and D.M.; methodology, E.D., D.T.; software, MA.L.; validation, D.M., E.D. and F.P.; formal analysis, MA.L. and E.D.; investigation, MA.L.; resources, MA.L., D.T.; data curation, E.D., D.M.; writing—original draft preparation, E.R.; writing—review and editing, E.D., E.R.; visualization, D.M., F.P.; supervision, F.P.; project administration, F.P. All authors have read and agreed to the published version of the manuscript.

## Guarantor

Ettore Dinoto.

## Research registration number

Not applicable

## Declaration of competing interest

The authors declare no conflict of interest.

## Data Availability

The data presented in this study are available on request from the corresponding author.
